# CXCL5 impedes CD8^+^ T cell immunity by upregulating PD-L1 expression in lung cancer *via* PXN/AKT signaling phosphorylation and neutrophil chemotaxis

**DOI:** 10.1186/s13046-024-03122-8

**Published:** 2024-07-22

**Authors:** Dantong Sun, Lipin Tan, Yongbing Chen, Qiang Yuan, Kanqiu Jiang, Yangyang Liu, Yuhang Xue, Jinzhi Zhang, Xianbao Cao, Minzhao Xu, Yang Luo, Zhonghua Xu, Zhonghen Xu, Weihua Xu, Mingjing Shen

**Affiliations:** 1 Department of Thoracic and Cardiac Surgery, The Second Affiliated Hospital of Soochow University, Suzhou, 215004 China; 2 Department of nursing administration, The Second Affiliated Hospital of Soochow University, Suzhou, 215004 China; 3 Department of interventional medicine, The Second Affiliated Hospital of Soochow University, Suzhou, 215004 China; 4 Department of Vascular Surgery, Hospital of Zhangjiagang, Suzhou, 215600 China; 5 Department of Thoracic Surgery, Hospital of Yancheng, Yancheng, 224000 China

**Keywords:** CXCL5, T cell immunity, PD-L1, PXN/AKT signaling, Neutrophils

## Abstract

**Background:**

Lung cancer remains one of the most prevalent cancer types worldwide, with a high mortality rate. Upregulation of programmed cell death protein 1 (PD-1) and its ligand (PD-L1) may represent a key mechanism for evading immune surveillance. Immune checkpoint blockade (ICB) antibodies against PD-1 or PD-L1 are therefore widely used to treat patients with lung cancer. However, the mechanisms by which lung cancer and neutrophils in the microenvironment sustain PD-L1 expression and impart stronger inhibition of CD8^+^ T cell function remain unclear.

**Methods:**

We investigated the role and underlying mechanism by which PD-L1^+^ lung cancer and PD-L1^+^ neutrophils impede the function of CD8^+^ T cells through magnetic bead cell sorting, quantitative real-time polymerase chain reaction (RT-PCR), western blotting, enzyme-linked immunosorbent assays, confocal immunofluorescence, gene silencing, flow cytometry, etc. In vivo efficacy and safety studies were conducted using (Non-obeseDiabetes/severe combined immune deficiency) SCID/NOD mice. Additionally, we collected clinical and prognostic data from 208 patients who underwent curative lung cancer resection between 2017 and 2018.

**Results:**

We demonstrated that C-X-C motif chemokine ligand 5 (CXCL5) is markedly overexpressed in lung cancer cells and is positively correlated with a poor prognosis in patients with lung cancer. Mechanistically, CXCL5 activates the phosphorylation of the Paxillin/AKT signaling cascade, leading to upregulation of PD-L1 expression and the formation of a positive feedback loop. Moreover, CXCL5 attracts neutrophils, compromising CD8^+^ T cell-dependent antitumor immunity. These PD-L1^+^ neutrophils aggravate CD8^+^ T cell exhaustion following lung cancer domestication. Combined treatment with anti-CXCL5 and anti-PD-L1 antibodies significantly inhibits tumor growth in vivo.

**Conclusions:**

Our findings collectively demonstrate that CXCL5 promotes immune escape through PD-L1 upregulation in lung cancer and neutrophils chemotaxis through autocrine and paracrine mechanisms. CXCL5 may serve as a potential therapeutic target in synergy with ICBs in lung cancer immunotherapy.

**Supplementary Information:**

The online version contains supplementary material available at 10.1186/s13046-024-03122-8.

## Introduction

Lung cancer remains the most prevalent cancer globally and the leading cause of cancer-related death. Over 2 million new diagnoses and 1.7 million deaths are reported annually, with numbers continuing to rise [1]. Approximately 85% of the diagnosed lung cancer cases are non-small cell lung cancer (NSCLC), with adenocarcinoma and squamous carcinoma comprising over 50% and 30% of the cases, respectively. Recently, immune checkpoint blockade (ICB) therapies targeting programmed cell death protein 1 (PD-1) and its ligand 1 (PD-L1) have shown considerable efficacy in clinical settings. Of note, PD-1/PD-L1 blocking antibodies enhance endogenous antitumor immunity, greatly benefiting a subset of patients with lung cancer through ICB [2, 3]. However, most patients exhibit poor responses to ICBs due to distinct primary or adaptive resistance that develops during treatment [4, 5]. Therefore, further understanding of the molecular mechanisms underlying PD-L1 expression in lung cancer is necessary for improving the clinical effect of PD-L1/PD-1 therapy [6, 7].

Chemokines are small, secreted proteins that signal through cell surface G protein-coupled chemokine receptors. They are best known for their ability to promote the migration of cells, most notably leukocytes. Consequently, chemokines play a central role in the development and homeostasis of the immune system, influencing either protective or destructive immune responses. Accumulating evidence validates the role of chemokines in regulating the cancer-immunity cycle of lung cancer. For instance, C-C motif ligand 7 (CCL7) recruits dendritic cells to promote antitumor immunity and facilitate anti-PD-1 immunotherapy by promoting T cell expansion [8]. Conversely, high expression of CCL5 correlates with poor prognosis, accumulation of immunosuppressive regulatory T cells (Tregs), and impaired CD8 effector function in patients with lung adenocarcinoma [9]. In addition, C-X-C motif chemokine ligand 9 (CXCL9) and CXCL10 are critical for CD8^+^ T cell infiltration into the tumor site to facilitate a productive antitumor response [10].

Among all chemokines, CXCL5 stands out as one of the most important in the tumor microenvironment (TME). Indeed, various malignant tumor types, including NSCLC [11], exhibit high levels of CXCL5 expression compared to para-carcinoma or normal tissues. CXCL5 binds to its receptor, C-X-C motif chemokine receptor 2 (CXCR2), and the CXCL5/CXCR2 axis directly promotes angiogenesis, tumor growth, and metastasis *via* the ERK/Snail pathway or the AKT/GSK3β/β-catenin pathway [12–15]. Importantly, CXCL5 has been shown to exert chemotactic effects on diverse immune subsets, such as neutrophils, myeloid-derived suppressor cells (MDSCs), and macrophages [16, 17]. Blockade of the CXCL5/CXCR2 signaling axis can increase the sensitivity of immunotherapy and delay tumor progression. However, the biological implications of CXCL5 in regulating PD-1/PD-L1 signaling and antitumor immunity in lung cancer remain largely unclear.

In this study, we observed marked overexpression of CXCL5 in lung cancer cells, which compromised CD8^+^ T cell-dependent antitumor immunity. We further elucidated that high PD-L1 expression in cancer cells resulted from enhanced phosphorylation of the Paxillin (PXN)/AKT signaling pathway triggered by CXCL5 stimulation of CXCR2 in a dose-dependent manner. Moreover, this CXCL5-*p*-PXN/AKT-PD-L1 signaling cascade constituted a positive feedback loop. Given the considerable expression of CXCR2 in neutrophils, we also investigated the paracrine role of CXCL5 in neutrophils. CXCL5-treated neutrophils further increased PD-L1 expression in lung cancer cells by releasing (granulocyte-macrophage colony-stimulating factor (GM-CSF). Moreover, we demonstrated that PD-L1^+^ neutrophils, after co-culture with lung cancer cells, exacerbated the CD8^+^ T cell exhaustion process. In addition, we investigated the influence of CXCL5 on the survival of patients with lung cancer, which revealed a positive correlation between CXCL5 levels and poor prognosis. Consistently, the combination of anti-CXCL5 and anti-PD-L1 treatment significantly inhibited tumor growth in vivo. Our findings collectively demonstrate that CXCL5 overexpression promotes immune escape and predicts a poor outcome in patients with lung cancer, indicating that CXCL5 may serve as a potential therapeutic target that could synergize with ICBs.

## Materials and methods

### Human samples

A total of 208 patients diagnosed with lung cancer at the Second Affiliated Hospital of Soochow University between 2017 and 2018 were enrolled in this study. Paraffin-embedded tumor tissues were collected from the Department of Pathology. None of the patients in this study received systemic treatment before sample collection. Patients were followed up for survival or lung cancer-related death every three months for five successive years. The study was approved by the Medical Ethics Committee of the Second Affiliated Hospital of Soochow University, and written informed consent was obtained from all patients prior to participation.

### Cell culture

Human lung cancer cells, including lung adenocarcinoma (LAUD) and Lung squamous cell carcinoma (LUSC) (A549 and H226), and the normal lung epithelial cell line BEAS-2B were obtained from the Cell Bank of the Chinese Academy of Sciences (Shanghai, China). A549 and H226 cells were cultured in RPMI-1640 medium supplemented with 10% fetal bovine serum (FBS) and 1% penicillin at 37℃. All cells were authenticated using the short tandem repeat method and were checked for mycoplasma contamination.

### Isolation of tumor-infiltrating neutrophils (TINs) from lung cancer tissues

Fresh lung cancer tissues were washed three times with (phosphate buffer saline (PBS)containing 1% FBS before being minced into small pieces. The specimens were collected in RPMI-1640 medium containing collagenase IV (1 mg/mL) and deoxyribonuclease I (10 mg/mL) and dissociated using the MACS Dissociator (Miltenyi BioTech). Dissociated cell suspensions were further incubated for 1 h at 37℃ under continuous rotation. The cell suspensions were then filtered through a 40-µm cell strainer (Biologix). TINs were purified using anti-Custer of differentiation 66b (CD66b) magnetic beads (Miltenyi BioTech). The purity of the TINs was evaluated by flow cytometry using an anti-CD66b antibody and exceeded 90%.

### Isolation of neutrophils and CD8^+^ T cells from peripheral blood mononuclear cells (PBMCs)

PBMCs from healthy donors were isolated by density-gradient centrifugation using Ficoll-Paque Plus. Blood neutrophils were harvested after the lysis of red blood cells using a lysis solution. CD66b^+^ neutrophils and CD8^+^ T cells were purified from PBMCs using anti-CD66b (Miltenyi Biotec) and anti-CD8 (Miltenyi BioTech) magnetic beads. The sorted cells were used unless their purity exceeded 90%.

### In vitro neutrophil-CD8 ^+^ T cell co-culture system

In a three-day incubation, bead-purified peripheral CD8^+^ T cells (1 × 10^5^ cells/well in 96-well plates) were co-cultured with A549 or H226 “educated” neutrophils isolated from PBMCs. For co-stimulation experiments, 96-well flat-bottom culture plates were coated overnight with anti-CD3 antibody (10 µg/mL, Abcam). Neutrophils and CD8^+^ T cells were cultured at a 2:1 ratio in 200 µL RPMI-1640 medium containing rIL-2 (20 IU/mL, Sino Biological), anti-CD3 (10 µg/mL, Abcam), and anti-CD28 (2 µg/mL, Abcam) antibodies, with or without a human anti-PD-L1 neutralizing antibody (4 µg/mL, Abcam). When indicated, co-culture experiments were performed using Transwell plates with a pore size of 0.4 μm (Corning). After the three-day incubation period, the cells were harvested for further analysis.

### Cell transfection

To knock down (KD) specific target genes, we plated the cells at a density of 5 × 10^5^ cells/mL and transfected them with specific siRNA duplexes using the Lipofectamine 3000 Transfection reagent (Invitrogen) according to the manufacturer’s instructions. SiRNAs were provided by General Biol (Anhui, China). The oligonucleotide sequences of the siRNAs were as follows: Control siRNA: 5′-GGAGCGAGATCCCTCCAAAAT-3′, 3′-GGCTGTTGTCATACTTCTCATGG-5′; CXCL5 siRNA: 5′-CUGAAGAACGGGAAGGAAATT-3′, 3′-UUUCCUUCCCGUUCAGTT-5′; CXCR2 siRNA: 5′-CCUCAAGAUUCUAGCUAUATT-3′, 3′-UAUAGCUAGAAUCUUGAGGTT-5′; PD-L1 siRNA: 5′-TGGCATTTGCTGAACGCATTT-3′, 3′-TGCAGCCAGGTCTAATTGTTTT-5′.

### Flow cytometry

To assess apoptosis, we double-stained lung cancer cells with annexin V-FITC and propidium iodide (BD Biosciences, USA), with or without CD8^+^ T cell co-culture, following the manufacturer’s instructions and under specific experimental conditions. Staining was assessed using the Cyto-FLEX Flow Cytometer (Beckman Coulter, USA). To assess the proliferation of CD8^+^ T cells under different conditions, we performed flow cytometric analysis following standard protocols. CD8^+^ T cell suspensions, with or without neutrophils, were stained in vitro with fluorochrome-conjugated antibodies and matched-isotype control antibodies. Red cells were lysed with an ammonium chloride solution, and samples were incubated with the Live/Dead Fixable Dead Cell Staining Kit (Invitrogen) prior to staining to allow for the identification of live cells. The stained cells were subsequently analyzed by multicolor flow cytometry.

### Quantitative real-time PCR (qRT-PCR)

Total RNA was extracted from lung cancer cells using the Trizol reagent (Invitrogen), and first-strand cDNA was reversed-transcribed using the All-in-One cDNA Synthesis SuperMix (E047-01 A, Novoprotein, China). qPCR was performed using the Advanced SYBR Green Supermix and the CFX Connect RT System (BioRad) to examine gene expression. The data obtained for each gene were normalized to the expression of GAPDH. The primer sequences used were as follows: GAPDH: forward, 5′-GAGAAGTATGACAACAGCCTCAA-3′ and reverse, 3′-GCCATCACGCCACAGTTT-5′; PD-L1: forward, 5′-GGAAATTCCGGCAGTGTACC-3′.

and reverse, 3′-GAAACCTCCAGGAAGCCTCT-5′; CXCL5: forward, 5′-CAATCTTCGCTCCTCCAATC-3′ and reverse, 3′-CTCCTTGCGTGGTCTGTAAA-5′; CXCR2: forward, 5′-ACACGCACACTGACCCAGAA-3′ and reverse, 3′-CGTGAATCCGTAGCAGAACA-5′.

### Western blot analysis

Lung cancer cells were lysed using the RIPA buffer on ice for 10 min. The lysates were centrifuged at 12,000 × *g* for 10 min at 4℃, and the supernatants were collected for protein concentration determination. Total proteins were separated on a 10% SDS-PAGE gel and transferred to a PVDF membrane (Millipore). The blot was incubated with appropriate primary antibodies at 4℃ overnight. Proteins were quantified through densitometric analysis and normalized to GAPDH.

### Enzyme-linked immunosorbent assays (ELISA)

Chemokines and cytokines produced by lung cancer cells or neutrophils were detected using human chemokine and cytokine ELISA kits (EK158, EK180, EK182; MultiSciences, China) following the manufacturer’s instructions.

The sections were deparaffinized with xylene, rehydrated in 100%, 85%, and 70% ethanol for 10 min, quenched for endogenous peroxidase activity with 3% hydrogen peroxide, and subjected to antigen retrieval in 0.5 mM EDTA buffer (pH 8.0) by heating in a microwave for 20 min. The sections were allowed to cool naturally to room temperature and then stained with various antibodies diluted in PBS containing 1% BSA, followed by incubation at room temperature for over 6 h. Immunostaining was performed, and subsequently, the sections were counterstained with hematoxylin for 5 min prior to coverslipping.

For H&E staining, mouse tissues were fixed with 4% paraformaldehyde, embedded in paraffin, fully dewaxed, hydrated, and stained with H&E. A random visual field was selected under a light microscope to observe the pathomorphological changes in each mouse tissue sample.

### Neutrophils and CD8 ^+^ T cell chemotaxis assay

A cell migration experiment was conducted using a Transwell system consisting of a polycarbonate membrane with a 5.0 μm pore size (5.0 μm, Corning). Neutrophils and CD8^+^ T cells were washed twice, resuspended in serum-free medium, and added to the top chamber, while conditioned medium was added to the bottom chamber. After 24 h of culture, the cells at the bottom of the chamber were collected and fixed with 4% paraformaldehyde. The number of neutrophils and CD8^+^ T cells that passed through the membrane was quantified using the Cyto-FLEX Flow Cytometer for 30 s.

### Confocal immunofluorescence

Frozen sections of tumor cells, neutrophils, and CD8^+^ T cells were fixed with 4% paraformaldehyde, followed by incubation with 5% goat serum for 1 h at room temperature to prevent nonspecific antibody binding. The sections were then incubated with primary antibodies overnight at 4℃. Secondary antibodies were used at a concentration of 5 µg/mL, followed by staining with DAPI. Stained sections were imaged using the LSM 880 Confocal Microscope (Zeiss, Jena, Germany).

**Tumor models and**in vivo**treatments**.

A549 cells were cultured, harvested, and suspended in PBS. For flank injections, a total of 0.2 mL containing 5 × 10^5^ cells were injected subcutaneously into the flank of six- to eight-week-old NOD/SCID mice (Hunan, China). The mice were housed in groups of five under specific pathogen-free conditions with unlimited access to food and water. Ten days after injection, the mice were treated with anti-CXCL5 and anti-PD-L1 antibodies. Tumor growth was assessed using the following formula: (length × width^2^)/2. All experimental protocols and animal care were approved by the Institutional Review Board of The Second Affiliated Hospital of Soochow University.

### Bioinformatics analysis

The Cancer Genome Atlas (TCGA) project, jointly initiated by the National Cancer Institute and the National Human Genome Research Institute in 2006, currently studies a total of 36 cancer types. TCGA utilizes large-scale sequencing-based genomic analysis technology, through extensive collaboration, to understand the molecular mechanisms of cancer, enhance scientific understanding of the molecular basis of cancer onset, and enhance our ability to diagnose, treat, and prevent cancer. Ultimately, it aims to create comprehensive maps detailing all changes in the cancer genome. We downloaded the RNA-seq data and survival characteristics data of patients with lung cancer from TCGA database for further research. Subsequent data analysis was conducted using R Studio 4.0.0. R Studio is a programming language and software environment with powerful data processing and analysis functions, including basic sequence analysis, molecular evolution, comparative genomics, protein structure comparison and prediction, and computer-aided drug design. Bioconductor, based on the R Studio environment, serves as a tool for visualizing, annotating, processing, analyzing, and collecting biological information, consisting of a series of R extension packages. The EdgeR and Ggplot2 R software packages were employed to identify genes with differential expression, using |log2FC| > 2 and FDR < 0.05 as selection criteria.

### Statistical analysis

All data are presented as means ± standard error of the mean (SEM), unless otherwise indicated. Statistical analyses were performed using a two-tailed Student’s *t*-test in GraphPad Prism software (GraphPad Inc., CA, USA), except for the analysis of the association of CXCL5 with the survival of patients with lung cancer, which utilized IBM SPSS Statistics 23.0. A p-value < 0.05 was considered statistically significant.

## Results

### Autocrine CXCL5 from lung cancer compromises antitumor immunity via PD-L1 upregulation and CD8^+^ T cell migration inhibition

**Fig. 1:**
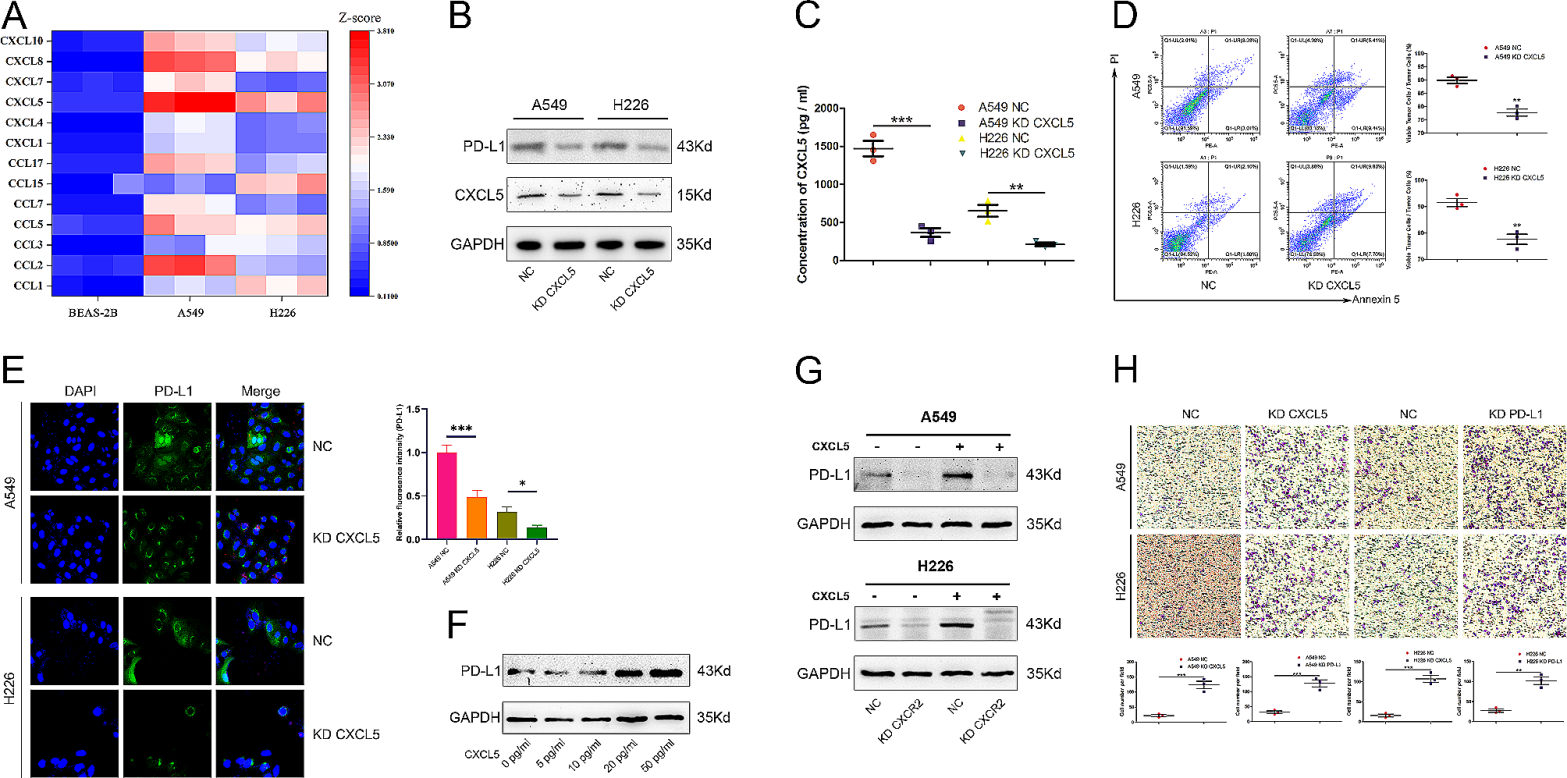
Autocrine CXCL5 from lung cancer compromises antitumor immunity *via* PD-L1 upregulation and inhibition of CD8^+^ T cell migration. (A) ELISA was performed to detect various chemokines secreted by BEAS-2B cells and lung cancer cell lines A549/H226. (B) The NC and KD-CXCL5 lung cancer cell lines A549/H226 were established through CXCL5 plasmid transfection, with CXCL5 levels examined by western blotting. (C) ELISA was performed to detect CXCL5 secretion by NC and KD-CXCL5 cells of A549/H226. (D) The apoptotic rates of NC and KD-CXCL5 A549/H226 cells were measured by flow cytometry for 6 h co-culture with CD8^+^ T cells (tumor cells: CD8^+^ T cells = 1:1). (E) Representative images of NC and KD-CXCL5 A549/H226 cells. Immunofluorescence was utilized to assess PD-L1 expression in each type of cell. (F) Western blotting was performed to analyze PD-L1 expression in response to CXCL5 in a dose-dependent manner. (G) To evaluate the autocrine effect of CXCL5, we established NC and KD-CXCR2 A549/H226 cell lines. PD-L1 expression was analyzed in NC and KD-CXCR2 A549/H226 cells with or without CXCL5 stimulation. (H) A Transwell assay was performed to examine the chemotaxis of CD8^+^ T cells to NC and KD-CXCL5 cells and NC and KD-PD-L1 A549/H226 cells, respectively. The data represent at least three independent experiments and are presented as the mean ± SEM. NS, not significant; *p < 0.05; **p < 0.01; ***p < 0.001

Initially, we investigated whether lung cancer cells produce distinct chemokine profiles compared to non-cancerous cells. To this end, we performed ELISA to detect various chemokines secreted by normal tracheal epithelial cells, BEAS-2B, and lung cancer cell lines, A549/H226. We found that CXCL5 secretion was significantly increased in lung cancer cells, as evidenced by heatmap analysis (Fig. 1A). Specific quantitative results of CXCL5 secretion by A549/H226 were also obtained (Supplementary Fig. 1A). To further investigate the biological functions of CXCL5 in lung cancer immunosurveillance, we established lung cell lines with either normal control(NC)or KD-CXCL5 using the Lipofectamine 3000 transient transfection method (Fig. 1C). The concentration of CXCL5 was determined through ELISA (Fig. 1B). Subsequently, we assessed the sensitivity of NC and KD-CXCL5 cells to CD8^+^ T cell-mediated killing. Flow cytometric analysis revealed that CXCL5 KD cells showed increased apoptosis when co-cultured with CD8^+^ T cells, indicating that CXCL5 KD could indeed enhance sensitivity to CD8^+^ T cell-mediated killing (Fig. 1D). Similarly, the application of IgG and anti-CXCL5 neutralizing antibodies showed consistent trends (Supplementary Fig. 1D). Notably, CXCL5 KD did not affect the apoptosis of lung cancers without co-culture with CD8^+^ T cells (Supplementary Fig. 1B).

Given the critical role of PD-L1 in mediating immune escape, we measured the expression of PD-L1 in NC A549 or H226 cells, as well as those cell lines with CXCL5 KD. Immunofluorescence staining showed a significant decrease in PD-L1 expression in lung cancer cells when CXCL5 expression was knocked down (Fig. 1E). Furthermore, we validated the dose-dependent increase in PD-L1 expression in response to CXCL5 (Fig. 1F). Subsequently, we characterized the role of CXCR2 in mediating PD-L1 expression. To this end, we established A549 or H226 cell lines with either control or CXCR2 siRNA (Supplementary Fig. 1E). Indeed, we found that CXCL5-triggered PD-L1 expression was blocked when CXCR2 expression was knocked down (Fig. 1G). The mRNA data is shown in Supplementary Fig. 1C. To further elucidate the role of the CXCL5-PD-L1 axis in mediating CD8^+^ T cell infiltration, we conducted a chemotaxis experiment and found that KD-CXCL5 or KD-PD-L1 indeed enhanced the chemotaxis of CD8^+^ T cells (Fig. 1H), which may represent an important mechanism regulating antitumor immunity in lung cancer. Conversely, the introduction of exogenous CXCL5 inhibited the chemotaxis of CD8^+^ T cells (Supplementary Fig. 1F).

### Positive feedback loop of the CXCL5-p-PXN/AKT-PD-L1 signaling cascade contributes to CD8^+^ T cell-mediated immune evasion by lung cancer cells

**Fig. 2:**
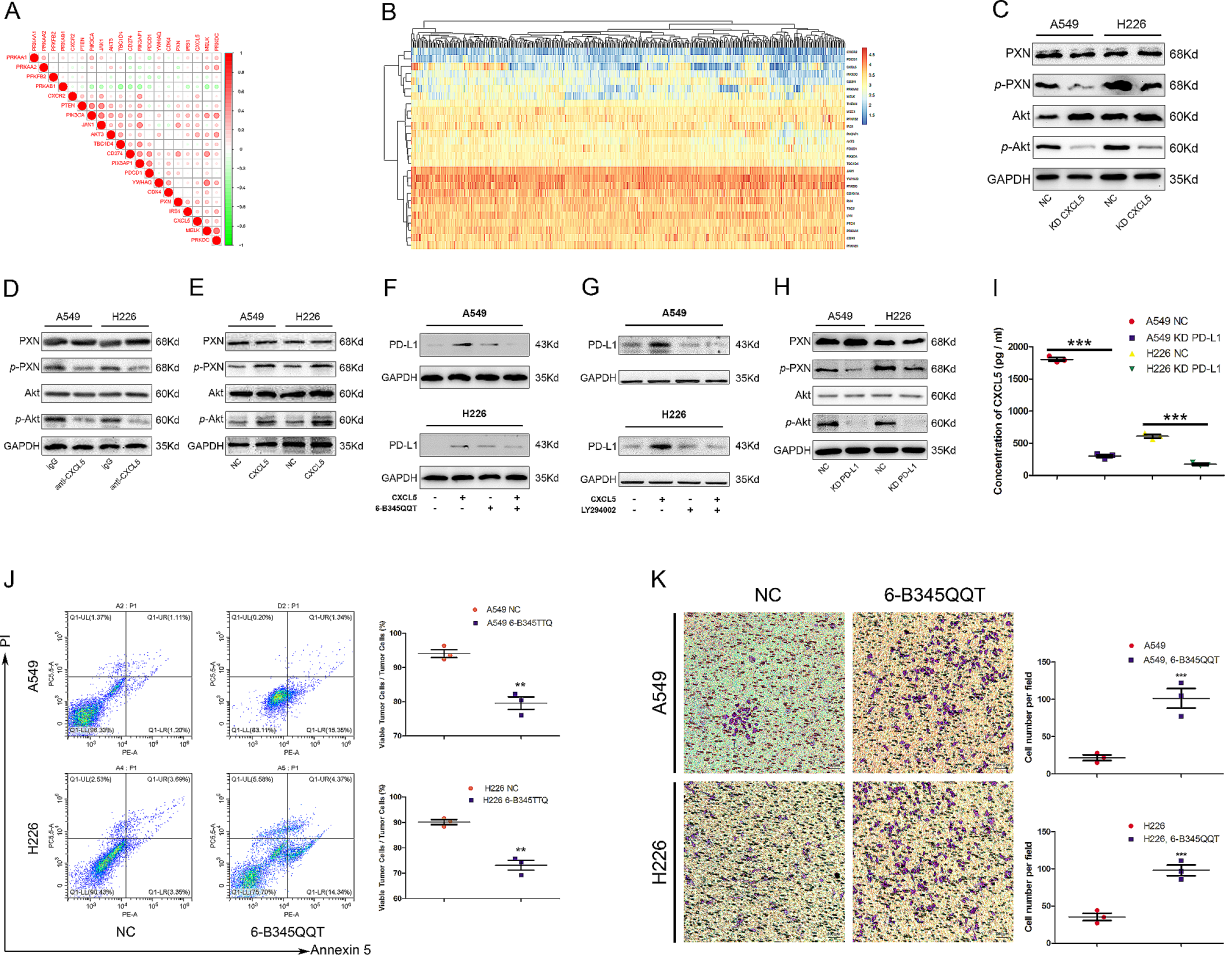
Positive feedback loop of the CXCL5-p-PXN/AKT-PD-L1 signaling cascade contributes to CD8^+^ T cell-dependent immunity escape in lung cancer cells. (A) Bioinformatics analysis using TCGA database revealed multiple genes associated with the CXCL5/PD-L1 pathway. (B) The heatmap displays clustering information for the correlated genes obtained through multinomial logistic regression. (C) Western blot was performed to analyze the activity and phosphorylation of the PXN/AKT pathway in NC and KD-CXCL5 cells. (D) Western blot was performed to analyze the activity and phosphorylation of the PXN/AKT pathway in cells treated with IgG and anti-CXCL5 antibodies. (E) Western blot was performed to analyze the activity and phosphorylation of the PXN/AKT pathway in NC and CXCL5-treated cells. (F, G) To demonstrate that PD-L1 was upregulated by CXCL5 stimulation *via* phosphorylation of PXN and AKT, we examined PD-L1 expression through western blotting with or without 6-B345TTQ and LYG294002 treatment under different CXCL5 conditions. (H) Phosphorylation of PXN and AKT in NC and PD-L1 KD A549/H226 cells was determined through western blotting. (I) CXCL5 concentrations in NC and PD-L1 KD lung cancer cells as determined through ELISA, which also decreased the phosphorylation of PXN/AKT in A549 and H226 cells. (J) The apoptotic rates of A549/H226 cells with or without 6-B345TTQ treatment were measured by flow cytometry for 6 h co-culture with CD8^+^ T cells (tumor cells: CD8^+^ T cells = 1:1). (K) A Transwell assay demonstrated that 6-B345TTQ treatment enhances the chemotaxis of CD8^+^ T cells. Data represent at least three independent experiments and are presented as the mean ± SEM. NS, not significant; * *p* < 0.05; **p < 0.01; ***p < 0.001

Bioinformatics analysis revealed that multiple genes are associated with the CXCL5/PD-L1 pathway. According to the cutoff criteria, 27 differentially expressed genes (DEGs) were identified (Fig. 2A). The heatmap(Fig. 2B) displayed that the genes *PRKDC*, *MELK*, and *PXN* were positively correlated with the expression of interacting genes, The results of the data statistics are shown in Supplementary Fig. 1G, wherein JAK/AKT/MELK and other genes are associated with PD-L1, according to known articles. To further characterize the regulatory relationship of CXCL5 and the PXN/AKT signaling pathway, we measured the activity of the PXN/AKT pathway through a western blotting assay. We found that CXCL5 KD significantly decreased the phosphorylation of both PXN and AKT in A549 or H226 cells (Fig. 2C). In addition, the application of a CXCL5 neutralizing antibody validated this finding (Fig. 2D), while CXCL5 exhibited the opposite trend (Fig. 2E). The small-molecule inhibitor 6-B345TTQ disrupted the interaction of PXN and α4 integrin, thereby interfering with α4 integrin signaling. CXCL5 stimulation did not improve PD-L1 expression upon 6-B345TTQ treatment (Fig. 2F). Together, the AKT inhibitor LY-294,002 exerted the similar effect (Fig. 2G). Notably, PD-L1 KD also decreased the phosphorylation of both PXN and AKT in A549 or H226 cells (Fig. 2H). Moreover, CXCL5 was measured in NC and PD-L1 KD lung cancer cells through ELISA (Fig. 2I) and IF (Supplementary Fig. 1K). In addition, flow cytometric analysis elucidated that inhibiting the PXN pathway with the small-molecule inhibitor 6-B345TTQ enhanced the killing of lung cancer cells by CD8^+^ T cells (Fig. 2J). As a control, 6-B345TTQ treatment did not exacerbate the apoptosis of lung cancer cells without CD8^+^ T cell co-culture (Supplementary Fig. 1I). Furthermore, a Transwell assay demonstrated that 6-B345TTQ treatment enhanced the chemotaxis of CD8^+^ T cells (Fig. 2K). In conclusion, CXCL5 promoted PD-L1 expression through the phosphorylated PXN/AKT pathway. Furthermore, KD-PD-L1 inhibited the phosphorylation of the PXN/AKT pathway and also inhibited the secretion of CXCL5, suggesting a positive feedback regulatory loop modulating CXCL5 or PD-L1 expression *via* the PXN and AKT signaling cascade. Additionally, survival analysis revealed that high PXN expression was associated with a poor prognosis in patients with lung cancer (Supplementary Fig. 1H).

### Neutrophil-derived GM-CSF induces the phosphorylation of the PXN/AKT pathway to promote PD-L1 expression in lung cancer

**Fig. 3:**
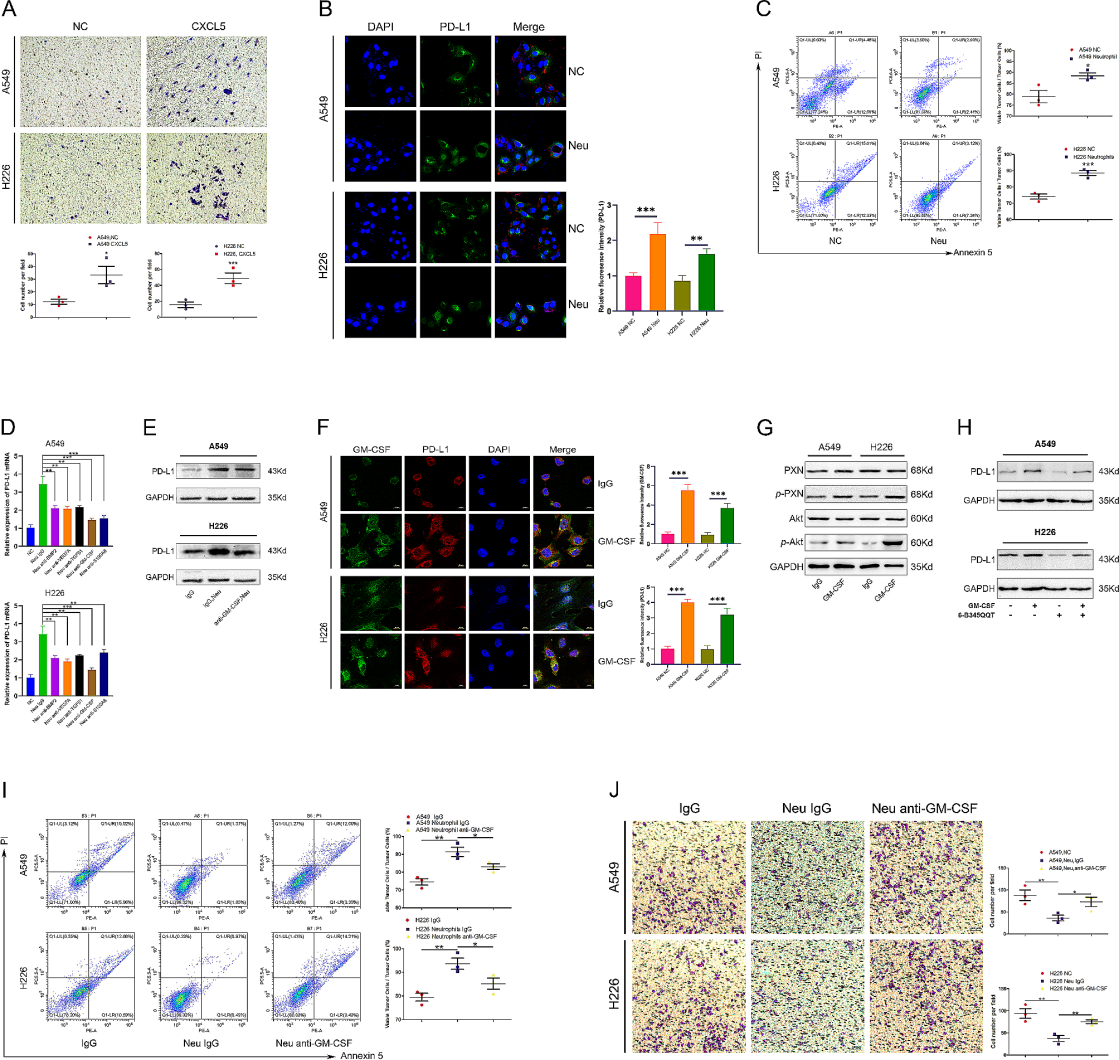
Neutrophil-derived GM-CSF induces the phosphorylation of the PXN/AKT pathway to promote PD-L1 expression in lung cancer. (A) A Transwell assay was performed to assess the chemotaxis of neutrophils with or without CXCL5 stimulation. (B) A549/H226 cells were co-cultured with or without neutrophils for 24 h, followed by immunofluorescence analysis of PD-L1 expression on A549/H226 cells. (C) A549/H226 cells were pretreated with or without neutrophils for 24 h and then co-cultured with CD8^+^ T cells. After 6 h, the apoptosis of A549/H226 cells was analyzed through flow cytometry. (D) RT-PCR was conducted to assess the mRNA expression of PD-L1 after treatment with neutrophils and antibodies against BMP2, VEGFA, TGFβ1, GM-CSF, and S100AB. (E) Western blot was performed to analyze PD-L1 expression in neutrophils and anti-GM-CSF antibody-treated cells. (F) Immunofluorescence was utilized to analyze the expression of PD-L1 on A549/H226 cells with or without stimulation of GM-CSF for 12 h. (G) After GM-CSF stimulation for 12 h, PXN and AKT signaling and phosphorylation in A549/H226 cells were examined through western blotting. (H) To demonstrate that GM-CSF upregulates PD-L1 *via* PXN and AKT phosphorylation, we assessed PD-L1 expression through western blotting with or without 6-B345TTQ treatment under different GM-CSF conditions. (I) A549/H226 cells were pretreated with or without neutrophils and anti-GM-CSF antibody for 24 h and then co-cultured with CD8^+^ T cells. After 6 h, the apoptosis of A549/H226 cells was analyzed through flow cytometry. (J) A Transwell assay demonstrated that anti-GM-CSF treatment can reverse the inhibition of chemotaxis in CD8^+^ T cells induced by neutrophils. Data represent at least three independent experiments and are presented as the mean ± SEM. NS, not significant; *p < 0.05; **p < 0.01; ***p < 0.001

Given that neutrophils express CXCR2, we hypothesized that lung cancer-secreted CXCL5 may attract neutrophils in peripheral blood mononuclear cells (PBMCs) to enter the TME (Fig. 3A). To this end, we established a co-culture system with neutrophils and lung cancer cells. The expression of PD-L1 in lung cancer significantly increased when co-cultured with neutrophils (Fig. 3B). Moreover, after co-culture of neutrophils and lung cancer cells, the killing of lung cancer cells by CD8^+^ T cells was inhibited, with more cancer cells surviving (Fig. 3C). Subsequently, we investigated the mechanism by which PD-L1 expression was upregulated in cancer cells co-cultured with neutrophils. Neutrophils are known to secrete various proteins, including GM-CSF, Bone morphogenic protein-2 (BMP2), Vascular endothelial growth factor A (VEGFA), Transforming growth factor-β1 (TGFβ-1), and S100A8, which are likely to regulate PD-L1. We found that GM-CSF had the most significant effect on promoting the expression of PD-L1 in lung cancer cells (Fig. 3D). The western blot data is shown in Fig. 3E. Immunofluorescence imaging analysis further validated that GM-CSF administration increased the expression of PD-L1 in lung cancer cells (Fig. 3F). The upregulation of PD-L1 by GM-CSF was concentration-dependent, as shown in Supplementary Fig. 1J. Notably, neutrophil-derived GM-CSF could also induce the phosphorylation of the PXN/AKT pathway to promote PD-L1 expression (Fig. 3G, H). Flow cytometric analysis validated the role of GM-CSF in mediating the killing of lung cancer cells by CD8^+^ T cells (Fig. 3I). Likewise, antagonizing neutrophil-derived GM-CSF could enhance CD8^+^ T cell chemotaxis (Fig. 3J).

### Lung cancer cell-educated PD-L1^+^ neutrophils promote CD8^+^ T cell exhaustion

**Fig. 4:**
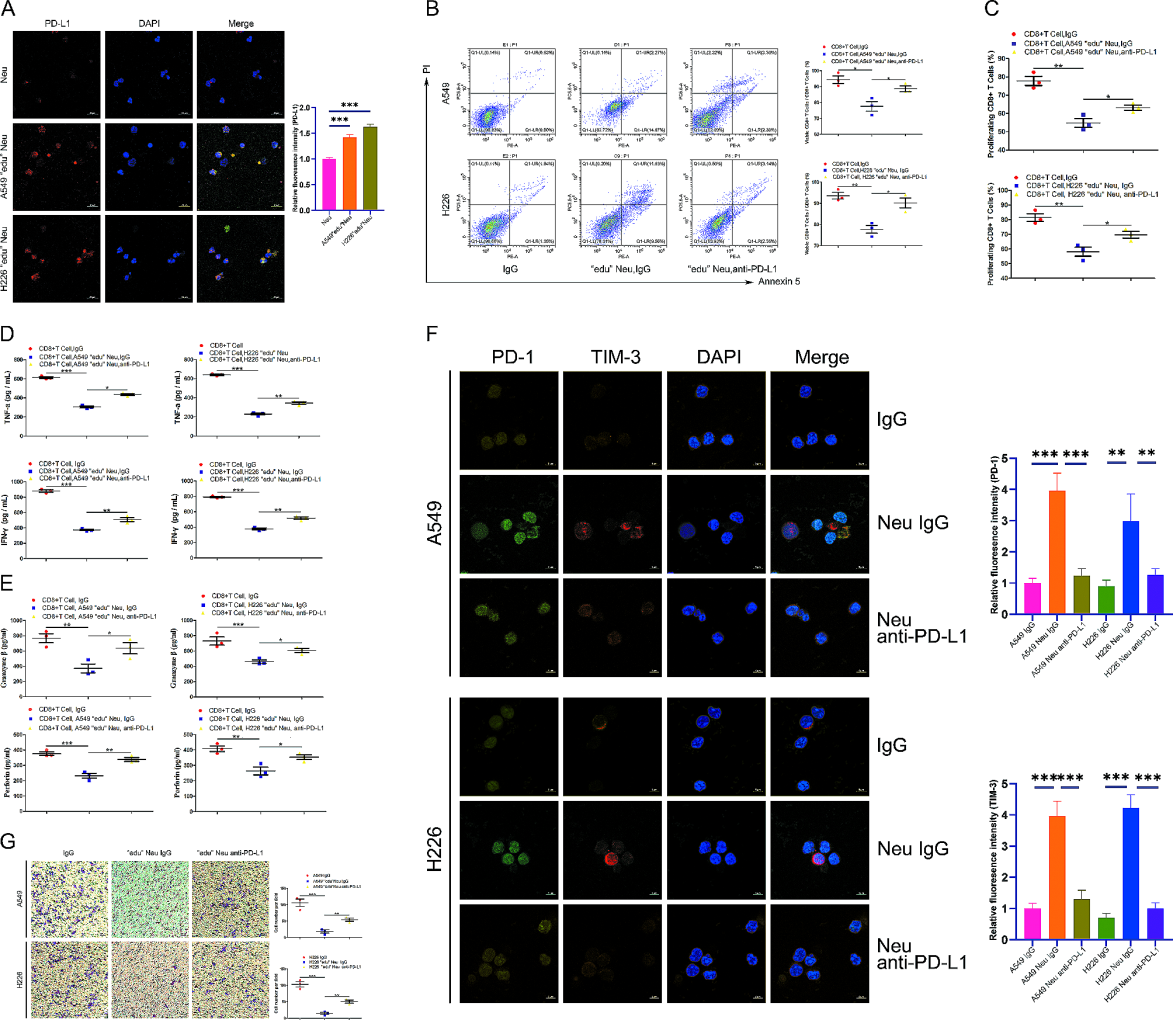
Lung cancer cell-educated PD-L1^+^ neutrophils promote CD8^+^ T cell exhaustion. (A) Immunofluorescence was utilized to analyze PD-L1 expression on neutrophils stimulated with or without A549/H226 cells for 24 h. Neutrophils were pretreated with or without anti-PD-L1 antibody for 12 h before co-culturing with CD8^+^ T cells for 72 h. Flow cytometry was conducted to analyze the apoptosis (B) and proliferation (C) of CD8^+^ T cells. ELISA was performed to assess the concentrations of TNF-α, IFN-γ (D), granzyme-β, and perforin (E) in CD8^+^ T cell media. Immunofluorescence was utilized to analyze the expression of PD-1 and TIM-3 on CD8^+^ T cells, with representative images shown (F). (G) A Transwell assay demonstrated that anti-PD-L1 treatment can reverse the inhibition of chemotaxis of CD8^+^ T cells induced by neutrophils. Data represent at least three independent experiments and are presented as the mean ± SEM. NS, not significant; *p < 0.05; **p < 0.01; *** *p* < 0.001

Lung cancer environments may contribute to the activated immunosuppressive phenotype of neutrophils. Consistent with our hypothesis, neutrophils significantly upregulated PD-L1 expression compared to non-co-cultured neutrophils (Fig. 4A). To further elucidate this effect, we isolated TINs from fresh lung cancer tissues. Compared to lung cancer-educated neutrophils from PBMCs, TINs expressed higher PD-L1 levels (Supplementary Fig. 2A). In addition to PD-L1, lung cancer cells significantly upregulated CD54, another granulocyte activation marker (Supplementary Fig. 2F). Subsequently, we investigated the potential impacts of recruited neutrophils on the functionality of CD8^+^ T cells. The presence of cancer-educated neutrophils significantly promoted the apoptosis of CD8^+^ T cells, which was partially rescued by blocking PD-L1 (Fig. 4B). We then measured the proliferation of CD8^+^ T cells (Fig. 4C). As expected, the proliferation of CD8^+^ T cells was severely impaired by cancer-educated neutrophils. The expression of diverse effector cytokines, including TNF-α, IFN-γ, granzyme-β, and perforin, was also suppressed by neutrophils. Blocking PD-L1 could, at least in part, rescue the decreased effector cytokine release (Fig. 4D, E). As controls, the proliferation, apoptosis, and the release of Tumor necrosis factor-α (TNF-α) and Interferon-γ (IFN-γ) by CD8^+^ T cells did not exhibit alterations following co-culture with “non-educated” neutrophils (Supplementary Fig. 2B–E). Collectively, these observations suggest that PD-L1 expression on neutrophils likely triggers a significant functional decline in CD8^+^ T cells. The expression of classical exhaustion markers PD-1 and TIM-3 also increased in the presence of PD-L1^+^ neutrophils, which reduced when PD-L1 signaling was blocked (Fig. 4F and Supplementary Fig. 2G). Furthermore, in the chemotaxis experiment, inhibition of PD-L1 signaling rescued the decreased chemotaxis of CD8^+^ T cells triggered by PD-L1^+^ neutrophils (Fig. 4G).

### Dual blockade of CXCL5 and PD-L1 inhibits lung cancer progression with good biological safety in vivo

**Fig. 5:**
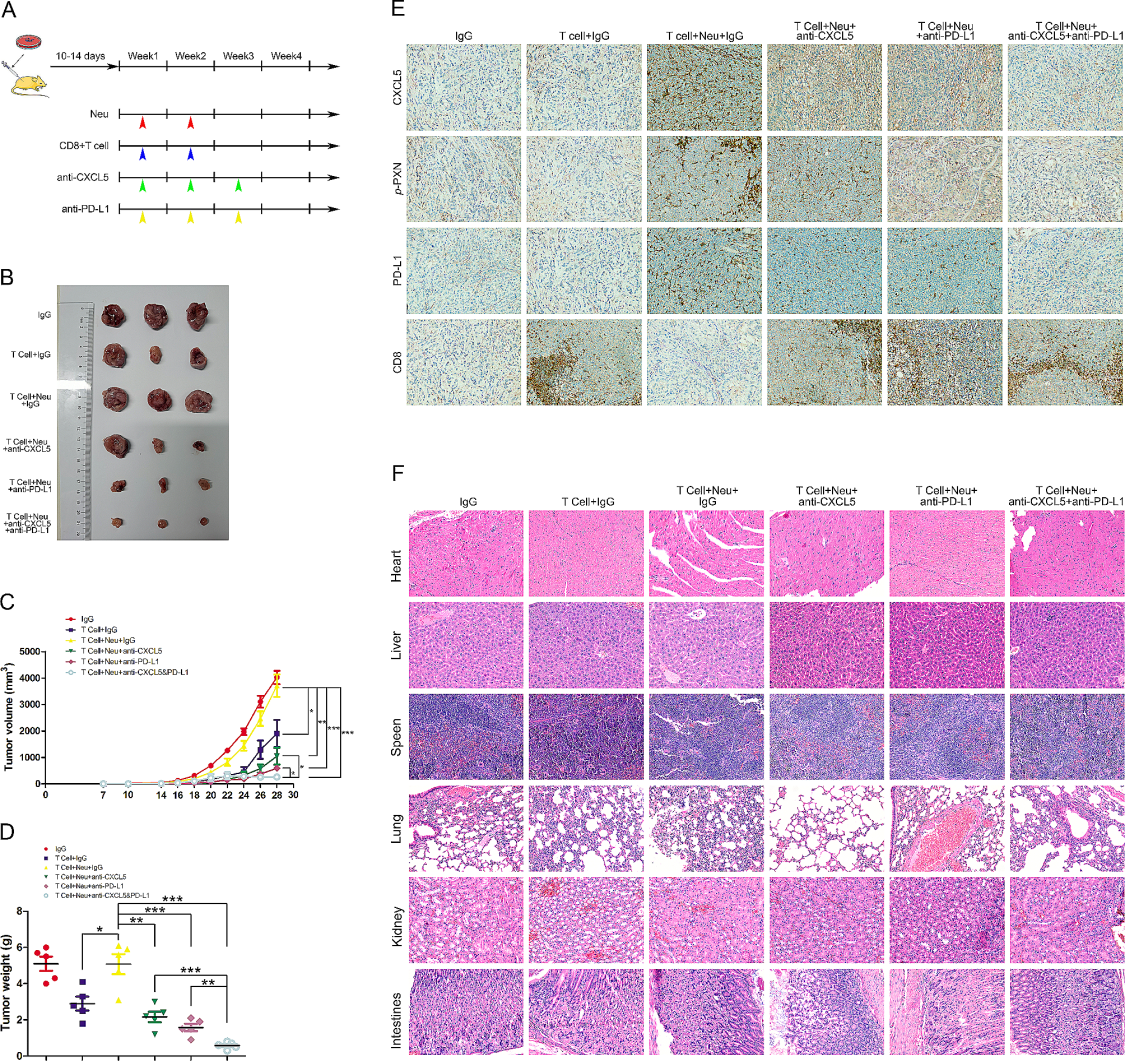
Dual blockade of CXCL5 and PD-L1 inhibits lung cancer progression with good biological safety in vivo. (A) A SCID/NOD mouse tumor-bearing model was employed to assess the CD8^+^ T cell-dependent antitumor effects of anti-CXCL5 and anti-PD-L1 neutralizing antibodies, with neutrophils and CD8^+^ T cells injected *via* the tail vein. The lung cancer cell line A549 was employed for tumor formation. (B) The tumors were excised and photographed six weeks after tumor cell injection. Tumor weight (C) and volume (D) calculations for each group are presented. (E) Representative images of immunohistochemical staining of CXCL5, *p*-PXN, PD-L1, and CD8 validating in vitro results. (F) H&E staining of heart, liver, lung, kidney, and intestinal tissues was conducted to assess the biological safety of anti-CXCL5 or anti-PD-L1 blockade antibodies. Data represent at least three independent experiments and are presented as the mean ± SEM. NS, not significant; *p < 0.05; **p < 0.01; ***p < 0.001

**Fig. 6:**
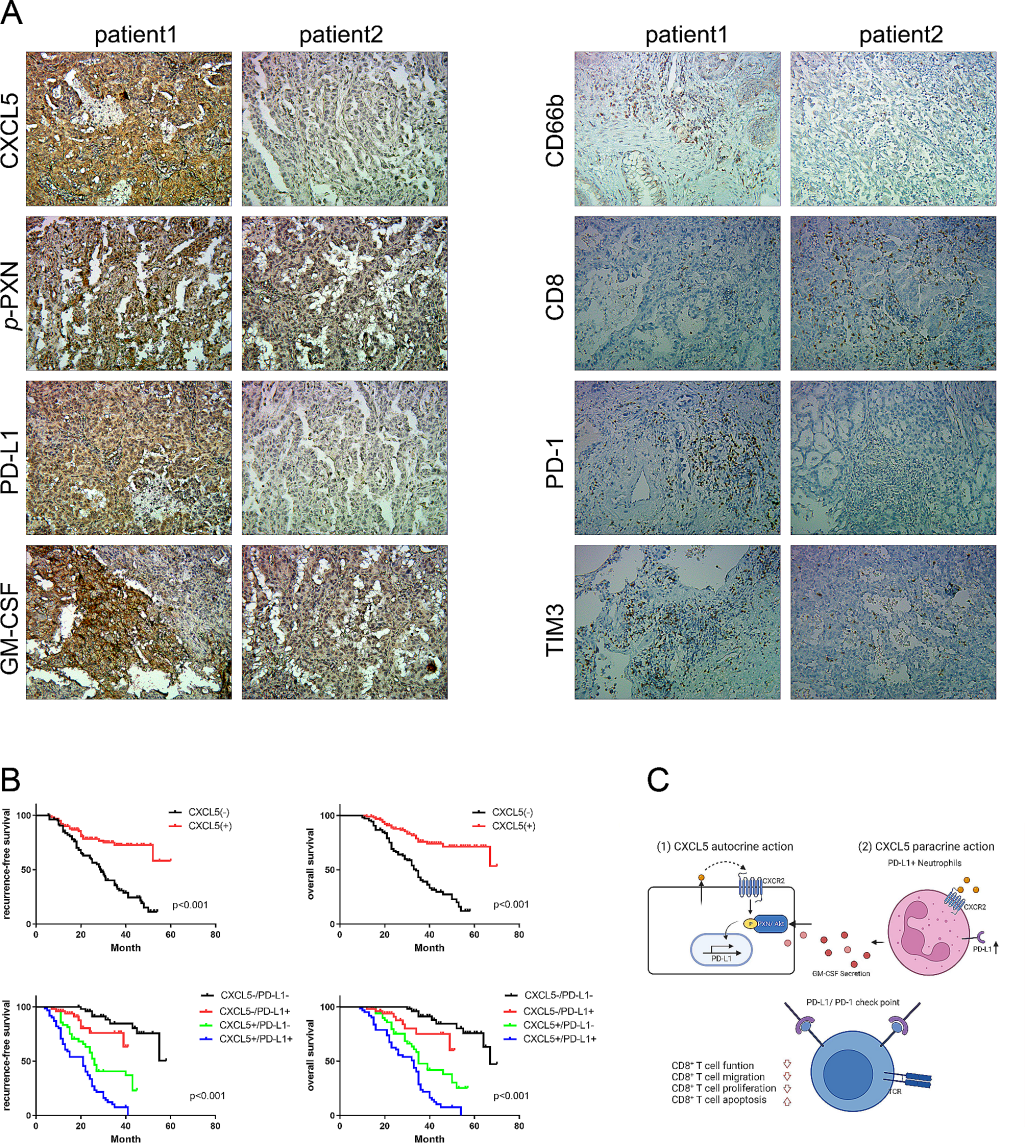
High CXCL5 and PD-L1 expression in lung cancer is associated with poor patient survival. Representative patients who underwent lung cancer resection were chosen from the cohort. (A) Immunohistochemical staining of CXCL5, *p*-PXN, PD-L1, GM-CSF, CD66b, CD8, PD-1, and TIM-3 from representative patients is shown. (B) The quantity of CXCL5 predicts the prognosis of patients with lung cancer, and the combined levels of CXCL5 and PD-L1 further predict the prognosis of patients with lung cancer. (C) Autocrine and paracrine effects of CXCL5 derived from lung cancer, leading to higher expression of PD-L1 on either lung cancer or neutrophils. Dual blockade of CXCL5 and PD-L1 inhibits lung cancer progression through CD8^+^ T cell immunity with good biological safety in vivo

We evaluated the potential therapeutic effects of dual blockade of CXCL5 and PD-L1 in vivo. For this purpose, the SCID/NOD mouse tumor-bearing model (Fig. 5A) was employed to assess the CD8^+^ T cell-dependent antitumor effects of CXCL5 and PD-L1 neutralizing antibodies. The SCID/NOD mice were injected with neutrophils and CD8^+^ T cells via the tail vein, followed by weekly injections of either anti-CXCL5 antibody or anti-PD-L1 antibody injections over a 3-week period subsequent to tumor formation using the A549 cell line. Representative tumors from each group are shown in Fig. 5B. As expected, adoptive transfer of T cells significantly delayed tumor growth (Fig. 5C), whereas the administration of neutrophils greatly reduced the tumor control of T cells. Of note, administration of blocking antibodies against either CXCL5 or PD-L1 improved therapeutic efficacy, with the combination of CXCL5 and PD-L1 blocking antibodies demonstrating superior tumor control. Moreover, similar results were observed in terms of distinct tumor weights (Fig. 6D). Consistently, the expressions of CXCL5, *p*-PXN, and PD-L1 decreased with CXCL5 or PD-L1 dual blockade, while the infiltration of CD8^+^ T cells was enhanced (Fig. 6E). Importantly, the administration of CXCL5 or PD-L1 blockade antibodies demonstrated good safety, as indicated by H&E staining of the heart, liver, lung, kidney, and intestinal tissues, which showed no obvious damage due to the treatment modality (Fig. 6F).

### High CXCL5 and PD-L1 expression in lung cancer is associated with poor patient survival

To elucidate the influence of key molecules on clinical outcomes, we performed immunohistochemical analysis of tumor tissue micro-arrays (TMAs) and quantified. In a cohort comprising primary tumors from 208 patients with lung cancer, we measured the number of CD66b^+^ neutrophils and the expression of CXCL5, *p*-PXN, PD-L1, GM-CSF, CD8, PD-1, and T cell immunoglobulin-3 (TIM-3). Representative cases are shown in Fig. 6A. Patients with lung cancer exhibiting higher CXCL5 expression displayed greater CD66b^+^ neutrophil infiltration, lower CD8^+^ T cell infiltration, and higher levels of *p*-PXN, PD-L1, GM-CSF, PD-1, and TIM-3 expressions. Conversely, patients with lung cancer exhibiting lower CXCL5 expression displayed less CD66b^+^ neutrophil infiltration, greater CD8^+^ T cell infiltration, and lower levels of *p*-PXN, PD-L1, GM-CSF, PD-1, and TIM-3 expressions. We performed survival analysis for high and low levels of CXCL5 and PD-L1 expressions and observed a significant association of high CXCL5 or PD-L1 expression with significantly decreased overall survival (OS) and recurrence-free survival (RFS) (OS: *p* < 0.001; RFS: *p* < 0.001) (Fig. 6B and Supplementary Fig. 2H). The log-rank tests revealed that patients with CXCL5^-^PD-L1^-^ exhibited the best prognoses, whereas patients with CXCL5^+^PD-L1^-^ or CXCL5^-^PD-L1^+^ demonstrated moderate prognoses, and patients with CXCL5^+^PD-L1^+^ exhibited the worst prognoses (OS: *p* < 0.01; RFS: *p* < 0.01) (Fig. 6B).

## Discussions

Tumor cells may employ multiple mechanisms to evade immune surveillance. In this study, we demonstrated the elevated expression of CXCL5 in lung cancer and explored its autocrine and paracrine roles. We found that high CXCL5 expression is associated with a poor prognosis and a decreased survival curve in lung cancer. In addition, combined utilization of anti-CXCL5 and anti-PD-L1 yielded the most effective outcome in controlling lung cancer, with convinced biological safety.

Chemokines play an instructive role in orchestrating the immune responses to tumors by influencing the cellular composition [18] and landscape of the TME [19, 20]. For instance, the production of CXCL9 and CXCL10 [21–23] is critical for attracting cytotoxic T cells and Th1 cells, which are required for mounting an effective antitumor immunity. Conversely, certain chemokines can recruit immunosuppressive subsets that facilitate tumor growth. For instance, CCL2 has been reported to recruit Tregs [24, 25], tumor-associated macrophages [26], as well as MDSCs [27, 28]. In particular, the CCL2/CCR2 axis has been well documented to mediate Treg recruitment and accumulation across gliomas [29] and diverse types of tumors [30]. In this regard, cancer cell-derived chemokines are crucial in shaping the infiltration pattern of either cancer-fighting effector cells or cancer-promoting immune subsets. Our study revealed that lung cancer cells generate high levels of CXCL5, promoting cancer escape or suppressing antitumor immunity through either autocrine or paracrine mechanisms. Upregulation of PD-L1 is a key mechanism mediating immune escape by binding to PD-1 on tumor-fighting CD8^+^ T cells. In this study, we demonstrated that tumor-secreted CXCL5 induces phosphorylation of the PXN/AKT pathway, leading to PD-L1 upregulation in lung cancer and enabling the evasion of immune surveillance by CD8^+^ T cells. Drawing on the understanding that MAPK/ERK signaling, JAK/STAT3 signaling, and interleukin-1b secretion [31–33] are implicated in PD-L1 expression, our group is the first to report the regulatory role of PXN/AKT phosphorylation in PD-L1 upregulation.

As one of the most abundant leukocytes in the immune system, accumulating evidence [34–36] suggests that neutrophils play an essential role in cancer progression and metastasis by releasing key inflammatory mediators, or cytokines. Of note, neutrophils have also been suggested to exert antitumor/antimetastatic effects in certain contexts [37]. Notably, neutrophils display a high degree of heterogeneity, displaying diverse activation states that elicit either antitumor or protumor functions. This is influenced by the distinct spectrum and levels of immunomodulatory mediators produced by cancer cells as well as cancer-associated cells within the TME. Our previous study as well as that of Antoine Schernberg [38, 39] demonstrated that neutrophils serve as a prognostic biomarker, indicating poor survival in locally advanced lung cancer. Neutrophils in the cancer environment can impede CD8^+^ T cell-dependent immune surveillance through various mechanisms, including Kras mutation, SETD2 deficiency-induced H3K36me3 methylation, miR-146a delivery, and the production of NO, GM-CSF, and arginase-1 [40–44]. Notably, we observed that lung cancer cells can induce the expression of PD-L1 in neutrophils, and these educated PD-L1^+^ neutrophils can notably impair CD8^+^ T cell functionality. Further investigations are required to elucidate the mechanism by which lung cancer cell-educated neutrophils trigger CD8^+^ T cell function exhaustion beyond the PD-L1/PD-1 signaling axis.

ICBs exert considerable therapeutic effects in a minority of patients in clinical settings [45, 46], highlighting the significant need to improve therapeutic efficacy through combined treatment modalities. Given the association between high CXCL5 expression levels and poor prognosis in patients with lung cancer and the autocrine and paracrine roles of CXCL5 in mediating notable immunosuppression and immune evasion, we investigated the combined treatment of lung cancer with dual blockade of CXCL5 and PD-L1. This approach was found to be safe and to improve CD8^+^ T cell antitumor immunity [47–50]. These findings may provide valuable insights for the development of novel therapeutic strategies aimed at improving response rates to ICBs through the concurrent blockade of CXCL5 and PD-L1 *in vivo.*

In summary, our study revealed that CXCL5 overexpression can upregulate PD-L1 *via* PXN/AKT phosphorylation in lung cancer. Furthermore, CXCL5 chemotactic neutrophils not only induce the release of GM-CSF, which activates the PXN/AKT signaling pathway, but also lead to CD8^+^ T cell functional exhaustion, ultimately inhibiting their antitumor ability. These findings, coupled with clinical sample analysis, suggest that CXCL5 may be a promising target for alleviating immunosuppression and could serve as a potential therapeutic target in synergy with ICBs.

## Conclusions

In this study, we found that autocrine CXCL5 by lung cancer improves its PD-L1 expression. In addition, paracrine CXCL5 attracts neutrophils into the lung cancer microenvironment. Mechanistically, CXCL5 activates the phosphorylation of the Paxillin/AKT signaling cascade, leading to upregulation of PD-L1 and the formation of a positive feedback loop. Furthermore, PD-L1^+^ neutrophils contribute to the progression of CD8^+^ T cell exhaustion. Inhibition of CXCL5-Paxillin/AKT-PD-L1 axis can reverse the CD8^+^ T cell depended immunity in vitro and in vivo. CXCL5 may serve as a potential therapeutic target in synergy with existed ICBs in lung cancer immunotherapy.

### Electronic supplementary material

Below is the link to the electronic supplementary material.

Supplementary Material 1

## Data Availability

Data are available upon reasonable request.

## Abbreviations

PD: 1 Programmed cell death protein 1
PD: L1 Programmed cell death protein ligand 1
ICB: Immune checkpoint blockade
CD8: Cluster of differentiation 8
RT-PCR: Quantitative real-time polymerase chain reaction
SCID/NOD: Non-obeseDiabetes/severe combined immune deficiency
CXCL5: C-X-C motif chemokine ligand 5
PXN: Paxillin
NSCLC: Non-small cell lung cancer
CCL7: C-C motif ligand 7
CCL5: C-C motif ligand 5
Tregs: Regulatory T cells
CXCL9: C-X-C motif chemokine ligand 9
TME: Tumor microenvironment
CXCR2: Chemokine receptor 2
MDSCs: Myeloid-derived suppressor cells
GM-CSF: Granulocyte-macrophage colony-stimulating factor
LAUD: Adenocarcinoma
LUSC: Lung squamous cell carcinoma
FBS: Fetal bovine serum
TINs: Tumor infiltrated neutrophils
CD66b: Custer of differentiation 66b
PBMCs: Peripheral blood mononuclear cells
KD: Knock down
TCGA: The Cancer Genome Atlas
SEM: Standard error of the mean
BMP2: Bone morphogenic protein-2
VEGFA: Vascular endothelial growth factor A
TGF-β1: Transforming growth factor-β1
TNF-α: Tumor necrosis factor-α
IFN-γ: Interferon-γ
TIM-3: T cell immunoglobulin-3
