# Supplementary material for: CXCL5 impedes CD8+ T cell immunity by upregulating PD-L1 expression in lung cancer via PXN/AKT signaling phosphorylation and neutrophil chemotaxis
Source: J Exp Clin Cancer Res. 2024 Jul 22;43:202. doi: 10.1186/s13046-024-03122-8 (PMC11264977; doi:10.1186/s13046-024-03122-8)

**Supplementary Figure 1** CXCL5/CXCR2-PXN-PD-L1 axis is implicated in CD8^+^ T cell-dependent immunity in lung cancer cells. (A) ELISA was performed to detect CXCL5 secretion by the lung cancer cell lines A549/H226. (B) Apoptotic rates of NC and KD-CXCL5 A549/H226 cells were measured by flow cytometry without CD8^+^ T cell co-culture. (C) Apoptotic rates of A549/H226 cells were measured after treatment with IgG and anti-CXCL5 antibodies without CD8^+^ T cell co-culture. (D) Western blot was performed to analyze CXCR2 expression in NC and KD-CXCR2 lung cancer cells. (E) RT-PCR was conducted to assess the mRNA expression of PD-L1 in NC and the KD-CXCR2 lung cancer cell lines A549/H226 with or without CXCL5 treatment. (F) A Transwell assay was performed to examine the chemotaxis of CD8^+^ T cells in A549/H226 cells treated with NC and CXCL5. (G) Detailed p-values from bioinformatics analysis using TCGA database revealed multiple genes associated with the CXCL5/PD-L1 pathway. (H) Immunofluorescence was utilized to analyze the expression of CXCL5 and PD-L1 in NC and KD-CXCL5 A549/H226 cells. (I) Apoptotic rates of NC and 6-B345QQT-treated A549/H226 cells were measured without CD8^+^ T cell co-culture. (J) The quantity of PXN predicts the prognosis of patients with lung cancer from TCGA database. (K) Western blot was performed to analyze PD-L1 expression in A549 in a dose-dependent manner. (L) Apoptotic rates of A549/H226 cells were measured with or without neutrophil co-culture and without CD8^+^ T cell co-culture. The data represent at least three independent experiments and are presented as the mean ± SEM. NS, not significant; *p < 0.05; **p < 0.01; ***p < 0.001.
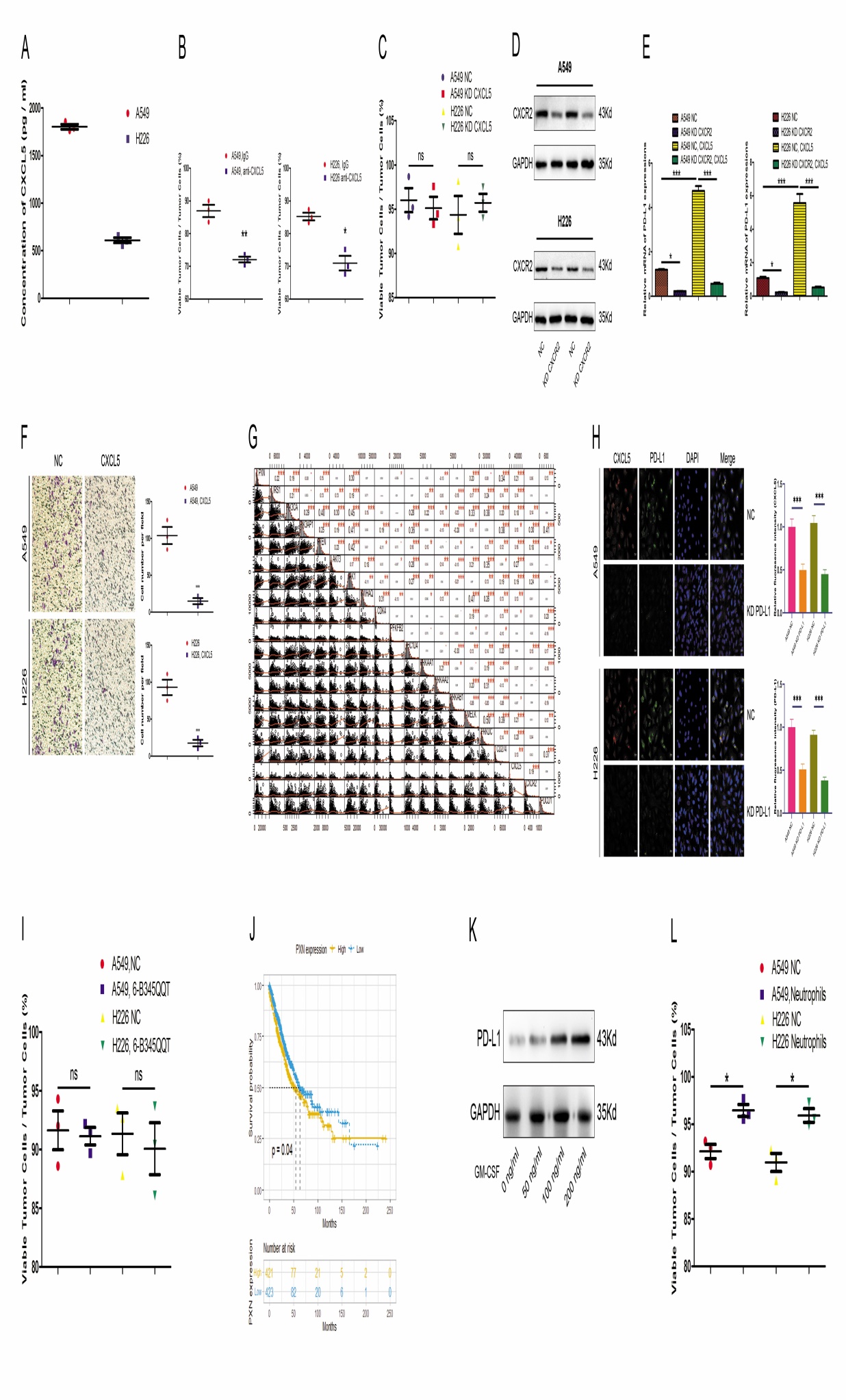


**Supplementary Figure 2** Lung cancer cell-educated PD-L1^+^ neutrophils promote CD8^+^ T cell exhaustion by activation. (A) Immunofluorescence was employed to analyze PD-L1 expression on lung cancer-stimulated neutrophils and tumor-infiltrating neutrophils. (B) Western blot was performed to analyze CD54 expression in neutrophils with or without A549/H226 co-culture.Flow cytometry was conducted to analyze the apoptosis (C) and proliferation (D) of CD8^+^ T cells with or without neutrophil co-culture. (E, F) ELISA was performed to assess the concentration of TNF-α and IFN-γ in CD8^+^ T cell media with or without neutrophil co-culture. (G) Immunofluorescence was employed to analyze PD-1 and TIM-3 expression on unstimulated neutrophils, PD-L1^-^ neutrophils, and PD-L1^+^ neutrophils. (H) The quantity of PD-L1 predicts the prognosis of patients with lung cancer. The data represent at least three independent experiments and are presented as the mean ± SEM. NS, not significant; *p < 0.05; **p < 0.01; *** p < 0.001.


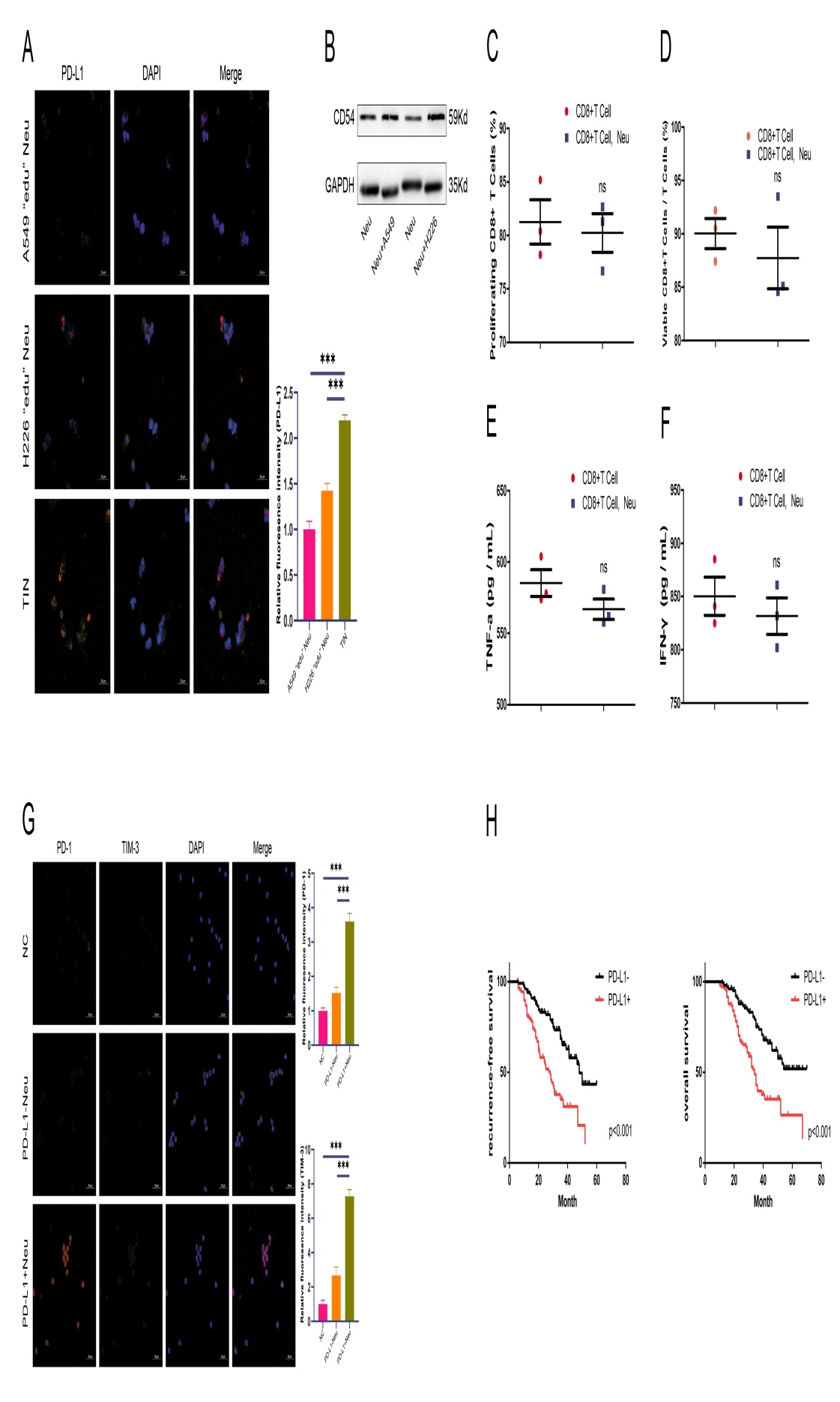

Supplement: Supplementary file 1 — Supplementary Material 1 [file 13046_2024_3122_MOESM1_ESM.docx]
